# Improving the Prediction of Total Surgical Procedure Time Using Linear Regression Modeling

**DOI:** 10.3389/fmed.2017.00085

**Published:** 2017-06-19

**Authors:** Eric R. Edelman, Sander M. J. van Kuijk, Ankie E. W. Hamaekers, Marcel J. M. de Korte, Godefridus G. van Merode, Wolfgang F. F. A. Buhre

**Affiliations:** ^1^Faculty of Health, Medicine and Life Sciences, Department of Health Services Research, CAPHRI School for Public Health and Primary Care, Maastricht University, Maastricht, Netherlands; ^2^Department of Clinical Epidemiology and Medical Technology Assessment (KEMTA), Maastricht University Medical Center+, Maastricht, Netherlands; ^3^Department of Anesthesiology, Maastricht University Medical Center+, Maastricht, Netherlands; ^4^Maastricht University Medical Center+, Maastricht, Netherlands

**Keywords:** operating room utilization, procedure time, regression, prediction, anesthesia time, surgeon time, surgical time

## Abstract

For efficient utilization of operating rooms (ORs), accurate schedules of assigned block time and sequences of patient cases need to be made. The quality of these planning tools is dependent on the accurate prediction of total procedure time (TPT) per case. In this paper, we attempt to improve the accuracy of TPT predictions by using linear regression models based on estimated surgeon-controlled time (eSCT) and other variables relevant to TPT. We extracted data from a Dutch benchmarking database of all surgeries performed in six academic hospitals in The Netherlands from 2012 till 2016. The final dataset consisted of 79,983 records, describing 199,772 h of total OR time. Potential predictors of TPT that were included in the subsequent analysis were eSCT, patient age, type of operation, American Society of Anesthesiologists (ASA) physical status classification, and type of anesthesia used. First, we computed the predicted TPT based on a previously described fixed ratio model for each record, multiplying eSCT by 1.33. This number is based on the research performed by van Veen-Berkx et al., which showed that 33% of SCT is generally a good approximation of anesthesia-controlled time (ACT). We then systematically tested all possible linear regression models to predict TPT using eSCT in combination with the other available independent variables. In addition, all regression models were again tested without eSCT as a predictor to predict ACT separately (which leads to TPT by adding SCT). TPT was most accurately predicted using a linear regression model based on the independent variables eSCT, type of operation, ASA classification, and type of anesthesia. This model performed significantly better than the fixed ratio model and the method of predicting ACT separately. Making use of these more accurate predictions in planning and sequencing algorithms may enable an increase in utilization of ORs, leading to significant financial and productivity related benefits.

## Introduction

Operating rooms (ORs) are some of the most valuable hospital assets there are, generating a large part of hospital revenue. Revenue per OR hour varies per procedure, but is estimated to be between $1,000 and $2,000 on average, before subtracting the variable costs of personnel and supplies related to hospitalization (1). This makes efficient utilization of ORs paramount. Every minute wasted may cause a significant loss of revenue. For efficient utilization of ORs, accurate schedules of assigned block time and sequences of patient cases need to be made.

The quality of these planning tools is dependent on the accurate prediction of total procedure time (TPT; abbreviations are described in Table 1) per case. TPT consists of anesthesia-controlled time (ACT, itself consisting of the induction and emergence phases) and surgeon-controlled time (SCT, being the duration of the actual operation, including patient positioning and draping). ACT is included because in Dutch academic hospitals, the induction and emergence phases always take place in the OR, making them relevant to OR utilization.

**Table 1:** Descriptions of abbreviations used.

ACT	Anesthesia-controlled time in minutes, as observed
Age	Patient age in years
ASA	American Society of Anesthesiologists physical status classification of the patient
eSCT	Surgeon-controlled time in minutes, as estimated prior to the operation
SCT	Surgeon-controlled time in minutes, as observed
TPT	Total procedure time in minutes, as observed

Predicted TPTs are used to plan up to a desired level of utilization of the OR complex. Sequencing patient cases based on predicted TPT can help minimize the probability of underutilization of the OR and cancelation of procedures. Previous research has shown that using a fixed ratio to calculate TPT from SCT as estimated prior to an operation [estimated surgeon-controlled time (eSCT)] provides more accurate estimates than adding a fixed duration for ACT to eSCT to compute TPT (2). In this paper, we attempt to improve the accuracy of TPT predictions further by including patient and surgery characteristics relevant to TPT.

## Materials and Methods

We extracted data from a Dutch benchmarking database of all surgeries performed in all eight academic hospitals in The Netherlands from 2012 till 2016. Written informed consent from the patients was not required, because no individual patient data were included. The data contributed by two of these hospitals were excluded, because they only contained observed and subsequently recorded SCT instead of the initially estimated SCT. The other records also did not contain eSCT, but did describe estimated TPT. We used this to approximate eSCT by subtracting 20 min, which is the default time allocated to ACT in many Dutch hospitals. Unfortunately, it was not feasible to accurately discover the exact time attributed to anesthesia for each operation in each hospital. Subtracting 20 min gives us approximate eSCTs that are sufficient for testing the methods described in this paper.

Potential predictors of TPT that were included in the subsequent analysis were eSCT, patient age, type of operation (identified by unique codes as registered by the hospitals), American Society of Anesthesiologists (ASA) physical status classification, and type of anesthesia used (again identified by hospital supplied codes). Other database fields described observed TPT, anesthesia induction time, and anesthesia emergence time. Observed ACT was calculated by adding up induction and emergence durations. Only records describing elective surgery were included, because emergency surgery does not receive an estimated TPT/SCT.

Data analysis and statistical calculations were performed in R version 3.3.1. Implausible or impossible data values, such as a 0 for observed TPT, were marked as missing data. As we suspected missing data in the database to have occurred completely at random, we omitted incomplete records from the analysis. The final dataset consisted of 79,983 records, describing 199,772 h of total OR time. The distribution of the characteristics within this dataset is shown in Tables 2 and 3. The data were split into a training set with records from the years 2012 till 2015 and a test set from 2016.

**Table 2:** Distribution of characteristics in the dataset.

Variable^a^		Percentage of dataset
Patient age	1–9^b^	9.1
	10–19	7.3
	20–29	7.6
	30–39	8.3
	40–49	12.3
	50–59	16.7
	60–69	20.5
	70–79	13.8
	80–89	4.2
	90–99	0.3
	100–103	<0.0
American Society of Anesthesiologists classification	1	32.9
	2	45.2
	3	20.4
	4	1.4
	5	<0.0
Main specialism	Ophthalmology	15.8
	Ear, nose, and throat	11.6
	Cardiothoracic surgery	10.6
	Orthopedic surgery	8.8
	Neurosurgery	8.4
	Plastic surgery	6.8
	Oral and maxillofacial surgery	4.5
	Obstetrics and gynecology	4.3
	Abdominal surgery	4.3
	Urology	4.0
	Surgical oncology	4.0
	Traumatology	3.5
	Obstetric and gynecological oncology	3.1
	Miscellaneous	2.7
	Pediatric surgery	2.1
	Vascular surgery	2.0
	Hepatobiliary surgery	1.4
	Transplant surgery	0.9
	Anesthesiology	0.8
	Pediatric gastroenterology	0.1

*Percentages are rounded to one decimal place*.

*^a^See Table 1 for the meaning of the abbreviations*.

*^b^Records describing patients with age 0 were omitted, because their number was so high that we suspected that users had entered 0 to indicate missing data*.

**Table 3:** Miscellaneous descriptive statistics about the dataset used.

Number of types of anesthesia described^a^	32
Number of types of surgery described^b^	4,458
Mean estimated total procedure time (TPT)	126 min
Median estimated TPT	90 min
Mean observed TPT	150 min
Median observed TPT	109 min
Mean observed anesthesia induction time	27 min
Median observed anesthesia induction time	23 min
Mean observed anesthesia emergence time	13 min
Median observed anesthesia emergence time	11 min

*Times are rounded to whole minutes*.

*^a^Type of anesthesia was based on the hospitals’ internal anesthesia codes. Examples of code meanings are “general,” “arterial line,” “epidural,” “spinal,” “local,” etc*.

*^b^Type of surgery was based on the hospitals’ procedure codes and descriptions. These precisely record the surgical procedures performed. As an example of the level of detail: there are different verification codes and descriptions for cataract surgery using different types of implanted lenses*.

An often used rule-of-thumb states the need for at least 10 records for each potential predictor of TPT to be included in the model. Recent research suggests the actual number may be even lower (3). Considering that the dataset used for our analysis contained nearly 80,000 records, we had ample precision to test all potential predictors and interactions.

First, we computed for each record the predicted TPT based on the fixed ratio model described by van Veen-Berkx et al. (2) For each patient, the eSCT was multiplied by 1.33. This number is based on the research performed by van Veen-Berkx et al., which showed that 33% of SCT is generally a good approximation of ACT. Using both predicted and observed TPT, we computed the mean absolute error (MAE), the mean squared error (MSE), and model fit expressed as the adjusted *R*-squared of the model. The adjusted *R*-squared can be interpreted as the proportion of variance in TPT that can be explained by parameters in the model.

All linear regression models were created using the 2012–2015 data and then validated on both this set and the 2016 set. This enabled us to separately measure the performance of the models on new data and compare this to their performance on the training data.

We used the *p*-value of each variable and the adjusted *R*-squared values to test all possible linear regression models to predict TPT using eSCT in combination with the other available independent variables.

As an additional alternative, all regression models were again tested without eSCT as a predictor to predict ACT separately (which leads to TPT by adding SCT). This allowed us to compare our findings with various previous attempts to predict ACT (4, 5).

Finally, to test for any possible influence, the omission of the incomplete records might have had on our results, we reran the analyses after imputation of the missing data. Linear regression was used to impute the numeric variables and a proportional odds model for the ordered variable describing ASA classification. The type of anesthesia used and the type of surgery performed could not be imputed, due to the large number of categories.

## Results

Using the fixed ratio model, the MAE of the 2012–2015 predictions was 39.5 min with a MSE of 3,859.6 min. For the 2016 predictions, the MAE was 38.5 min with a MSE of 3,275.9 min.

All variables of the linear regression models were highly significant predictors (*p* < 0.01), in part, due to the size of the dataset, except some of the levels of the factor variables for type of anesthesia and type of operation. These variables were retained in the model though, since the overall effect of the factor variables was significant. Ultimately, the best model was identified by examining when the adjusted *R*-squared showed only minimal improvement after adding additional predictors.

Of all models tested, TPT is most accurately predicted using a linear regression model based on all available independent variables. However, as can be seen in Tables 4 and 5, including patient age in the model did not significantly improve the goodness-of-fit, so we only retained the variables eSCT, type of operation, ASA classification, and type of anesthesia. Using this best model, the MAE of the 2012–2015 predictions was 29.2 min with a MSE of 2,320.7 min. For the 2016 predictions, the MAE was 31.3 min with a MSE of 2,366.9 min. The adjusted *R*-squared of this model was 0.8498.

**Table 4:** Goodness-of-fit of the linear regression models for predicting total procedure time ranked by best adjusted *R*-squared value.

Independent variable(s)^a^					Adjusted *R*-squared
Estimated surgeon-controlled time	Type of anesthesia	American Society of Anesthesiologists	Age	Type of operation	
+	+	+	+	+	0.8499
+	+	+		+	0.8498
+		+	+	+	0.8491
+	+			+	0.8491
+	+		+	+	0.8491
+		+		+	0.8490
+			+	+	0.8483
+				+	0.8483
+	+	+	+		0.7853
+	+	+			0.7852
+	+		+		0.7846
+	+				0.7843
+		+			0.7763
+		+	+		0.7763
+			+		0.7757
+					0.7756

*Models were based on the 2012–2015 data. Adjusted R-squared values are rounded to four decimal places*.

^a^See Table 1 for the meaning of the abbreviations.

**Table 5:** Goodness-of-fit of the linear regression models for predicting anesthesia-controlled time (ACT), ranked by best adjusted *R*-squared value.

Independent variable(s)^a^				Adjusted *R*-squared
Type of anesthesia	American Society of Anesthesiologists	Age	Type of operation	
+	+	+	+	0.6316
+	+		+	0.6314
+		+	+	0.6256
+			+	0.6246
	+	+	+	0.5991
	+		+	0.5988
			+	0.5925
		+	+	0.5925
+	+	+		0.3801
+	+			0.3677
+		+		0.3067
+				0.2561
	+	+		0.1346
	+			0.1346
		+		0.0162

*Models were based on the 2012–2015 data. Adjusted R-squared values are rounded to four decimal places*.

*^a^See Table 1 for the meaning of the abbreviations*.

*Models for predicting total procedure time included estimated surgeon-controlled time as independent variable, models for predicting ACT did not*.

Similarly, ACT was most accurately predicted by all independent variables, but with very little improvement by adding patient age. The final model, based on the type of operation, ASA classification, and type of anesthesia, did not perform better than the direct prediction of TPT, with a MAE of the 2012–2015 predictions of 34.7 with a MSE of 3,269.7 min and a MAE of the 2016 predictions of 34.2 min with a MSE of 2,878.7 min. The adjusted *R*-squared was 0.6314.

**Figure 1:**
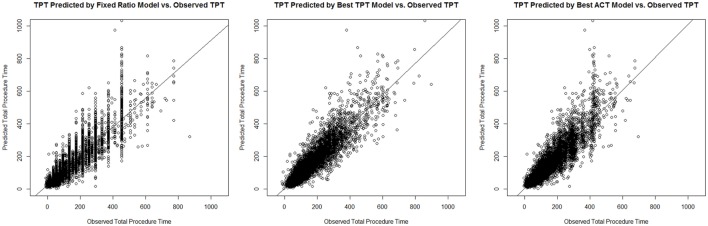
Plots of the predicted versus the actual total procedure time (TPTs) for the fixed ratio model and the two best linear regression models for predicting TPT and anesthesia-controlled time (ACT).

These main outcomes are summarized in Table 6. Figure 1 displays plots of the predicted versus the actual TPTs for these three models.

**Table 6:** Performance of fixed ratio model and best performing linear regression models.

	2012–2015		2016	
	Mean absolute error (MAE)	Mean squared error (MSE)	MAE	MSE
Fixed ratio model	39.5	3,859.6	38.5	3,275.9
Most accurate model for predicting total procedure time (TPT)^a^	29.2	2,320.7	31.3	2,366.9
Most accurate model for predicting anesthesia-controlled time (ACT)^b^	34.7	3,269.7	34.2	2,878.7

*Errors are the difference between predicted TPT and observed TPT in minutes and are rounded to one decimal place*.

*^a^Linear regression model based on the independent variables estimated surgeon-controlled time (eSCT), type of operation, American Society of Anesthesiologists (ASA) classification, and type of anesthesia*.

*^b^Linear regression model based on the independent variables type of operation, ASA classification, and type of anesthesia. TPT was predicted by adding predicted ACT to eSCT*.

After imputation of missing data in the initial dataset instead of elimination of incomplete records, all results were practically the same.

## Discussion

The improvement in TPT prediction of the best performing linear regression model versus the fixed ratio model was convincing. On the training data, the MSE was reduced by a quarter of the original value. This indicates that the variation in prediction errors was substantially reduced. As is to be expected, this effect was somewhat less pronounced on the 2016 testing data, but still very useful.

Making use of these more accurate predictions may help prevent the typical consequences of under- and overestimation. Underestimation can lead to costly overtime or even the cancelation of operations, while overestimation can lead to downtime of both the operating theater and its staff. For the hospital with the highest number of complete records in our dataset, totaling all the under- and overestimation of the included operations from 2016 results in a total overestimation of 3,118 h. Had they made use of a model as described in this paper (based on their own data), the total result would have been an overestimation of only 179 h. Depending on the way these hours would have been distributed in the scheduling, they may have led to additional operations being performed.

The accuracy of predicted durations of surgery also directly influences the confidence with which planners might increase the level of utilization of ORs. Planning for higher utilization is only possible with more certainty about case duration, but can offer significant financial and productivity related benefits.

A second important finding is that separate ACT prediction (using the same available variables but without eSCT) yields worse results than direct TPT prediction.

The fact that TPT is the result of ACT and SCT is demonstrated by the best performing model. This model is based on eSCT, type of operation, and the two most important anesthesiologic variables: ASA classification and type of anesthesia used. This means predictions are possible using a limited number of easily obtainable values. Even though our model is intended for use by a computer system, keeping the model simple by requiring fewer inputs improves its usability, understandability, and speed.

The fact that the regression models were calculated and tested using surgeons’ actual pre-surgery estimations of SCT instead of recorded, historical SCTs lends additional credibility to our results. In actual planning practice, predictions will similarly need to be based on estimated SCT. Therefore, the performance of the models as described in our results should match real-world performance, as opposed to a likely positive bias when based on historical data. This is especially true for the performance on the 2016 data, which the model was not trained on. While performing our research, it became apparent that the predictions of the 2016 TPTs became increasingly accurate as our collection of training data grew. This suggests that the method described in this paper holds potential for improved performance when applied to even larger datasets, as are becoming increasingly available to health-care data analysts. Additionally, further improvement may be achieved by tailoring the analyses to local circumstances. It is possible to prepare custom models for the level of individual hospitals, departments, types of operations, or even surgeons.

Summarizing the above, we encourage hospital data analysts and surgical managers to create similar models to those described in this paper using as much of their own historical data as possible. The method described is relatively straightforward and might provide them with more accurate procedure time predictions than current practices.

A limitation of this study was that the data used were recorded in academic centers only. The applicability to typical OR schedules in regional hospitals has not been studied. In addition, we have averaged all suitable data available from these academic centers under the assumption that there were no major differences between these centers that might significantly alter the TPT.

The manual registration of the timestamps and semi-manual process of aggregating the other data has two important weaknesses. First, it most probably resulted in inaccuracies of the data, possibly leaning toward late recording of the key moments during the operations. Second, there was a surprising amount of missing data at analysis. Of the records we started with, only ca. 21% contained complete and plausible data in all required fields, making the rest unsuitable for analysis. The fact that the results after imputation of the missing data were very similar to those of our initial analyses indicates that eliminating the incomplete records had limited influence on the outcomes as described.

Both issues underline the importance of the implementation of automatic registration systems that integrate into the work processes in the OR to collect more and better data. Only then will the results of analysis of this data be taken to a higher level, allowing for robust conclusions with operational consequences.

A final important remark is that, despite the new model generally performing well over the long-term, a relatively high interindividual variability still exists. This could limit the usefulness of its predictions in day to day planning.

## Conclusion

A linear regression model to predict TPT based on eSCT, type of operation, patient ASA classification, and anesthesia type outperforms the current practices of using a standard duration for ACT or a fixed ratio between eSCT and TPT. A second conclusion is that predicting TPT through the separate prediction of ACT yields less accurate results than direct prediction of TPT.

## Author Contributions

EE performed the analyses based on advice by SK and drafted the original manuscript. WB provided direct supervision during the entire project. AH, MK, and WB made valuable contributions to the anesthesiological aspects of the research performed and contributed to the article contents. GM independently performed the statistical analyses a second time to confirm the outcomes. He also provided additional advice and feedback on the methods and their textual descriptions.

## Conflict of Interest Statement

The authors declare that the research was conducted in the absence of any commercial or financial relationships that could be construed as a potential conflict of interest.
